# Detection and absolute quantification of *Lactiplantibacillus plantarum* ATCC 202195 by quantitative real-time PCR

**DOI:** 10.1128/spectrum.02711-23

**Published:** 2023-11-29

**Authors:** Jakaria Shawon, Lisa G. Pell, Mamun Kabir, Kara Evans, Mehedi Hasan, Grace Li, Huma Qamar, Cody W. E. Starke, Shakya Kurukulasuriya, Abdullah Al Mahmud, Philip M. Sherman, Shafiqul Alam Sarker, Daniel E. Roth, Rashidul Haque

**Affiliations:** 1 Nutrition and Clinical Services Division, International Centre for Diarrhoeal Disease Research, Bangladesh (icddr,b), Dhaka, Bangladesh; 2 Centre for Global Child Health, The Hospital for Sick Children, Toronto, Ontario, Canada; 3 Emerging Infections and Parasitology Laboratory, International Centre for Diarrhoeal Disease Research, Bangladesh (icddr,b), Dhaka, Bangladesh; 4 International Flavors & Fragrances Inc., Madison, Wisconsin, USA; 5 Cell Biology Program, Research Institute, The Hospital for Sick Children, Toronto, Ontario, Canada; 6 Department of Paediatrics, Faculty of Medicine, University of Toronto, Toronto, Ontario, Canada; National Research Council of Italy, Bari, Italy

**Keywords:** probiotics, bacterial detection, sepsis, qPCR

## Abstract

*Lactiplantibacillus plantarum* ATCC 202195 has probiotic properties that may be beneficial in early infancy. Since the effects of probiotics are often strain and dose specific, tools are needed to identify and enumerate *L. plantarum* ATCC 202195 with high sensitivity and specificity. Herein, a probe-based real-time quantitative PCR (qPCR) assay was developed to detect and enumerate *L. plantarum* ATCC 202195. The probe sequence contained a single nucleotide polymorphism within gene FEE41_08190 that was unique to *L. plantarum* ATCC 202195 at the time of the assay’s design. The linear dynamic range of the assay spanned five orders of magnitude with an *R* ^2^ that did not deviate from linearity and an amplification efficiency that ranged from 97% to 110% across six independent plates. At 95% confidence, the limit of detection in the assay was estimated at roughly 15 cells. No amplification was observed for reactions containing gDNA from seven other *Lactobacillus* species. This is the first report of a qPCR assay that detects and quantifies *L. plantarum* ATCC 202195. The assay is intended to be used as a resource for future clinical trials and commercial manufacturing settings.

**IMPORTANCE:**

When administered for seven consecutive days shortly after birth, the probiotic bacterium *Lactiplantibacillus plantarum* ATCC 202195 plus fructooligosaccharide (FOS) was reported to reduce sepsis and lower respiratory tract infection events during early infancy in a randomized trial in India. Since probiotic effects are often strain specific, strain-level detection and quantification by routine molecular methods enables the monitoring of safety outcomes, such as probiotic-associated bacteremia, and allows for the quality of probiotic interventions to be assessed and monitored (i.e., verify strain identity and enumerate). Despite the potential clinical applications of *L. plantarum* ATCC 202195, an assay to detect and quantify this strain has not previously been described. Herein, we report the design of primer and probe sequences to detect *L. plantarum* ATCC 202195 and the development and optimization of a real-time PCR assay to detect and quantify the strain with high specificity and high sensitivity.

## INTRODUCTION

*Lactiplantibacillus plantarum* ATCC 202195 is a probiotic that may improve early infant health outcomes. A community-based, placebo-controlled, randomized trial (*n* = 4,556) in India reported that a 7-day regimen of *L. plantarum* ATCC 202195, when orally administered as a synbiotic preparation combined with fructooligosaccharide (FOS) to neonates (birthweight ≥2,000 g and gestational age ≥35 weeks), reduced the number of episodes of clinical sepsis within the first 60 days of life, compared to a placebo, by 40% [95% confidence interval (CI) 0.48–0.74] (1). The same trial also reported significant reductions in the number of episodes of culture-negative [risk ratio (RR) 0.53, 95% CI 0.30–0.92] and culture-positive (RR 0.22, 95% CI 0.09–0.53) sepsis and lower respiratory tract infections (RR 0.66, 95% CI 0.51–0.88). Colonization of the infant gut by *L. plantarum* ATCC 202195 was not assessed in the trial. However, evidence from an earlier hospital-based trial (*n* = 31) of the same synbiotic regimen indicated that *L. plantarum* could be detected in infant stool after 3 days of supplementation (2) and was sustained for several months after supplementation ended (2). Of note, *L. plantarum* was identified in infant stool samples using conventional culture-based and biochemical methods rather than by molecular techniques. In addition, at the time when both trials were implemented and published, the whole genome sequence of *L. plantarum* ATCC 202195 was not yet publicly available.

Given the dramatic effects that *L. plantarum* ATCC 202195 had on infectious outcomes, there is interest in validating the results in a new setting and determining whether the results can be extended to infants who are born <2,000 g and at <35 weeks gestational age. There is also interest in determining the effect of the intervention on the gut microbiota and whether different supplementation regimens would be as efficacious at colonizing the infant gut (i.e., 1 day vs 7 days, with and without FOS). To address these knowledge gaps, we have implemented a phase 2 trial of neonatal oral supplementation of *L. plantarum* ATCC 202195 with or without FOS for 1 or 7 days in Dhaka, Bangladesh (Clinicaltrials.gov: NCT05180201). The investigational product for the trial was manufactured by International Flavors & Fragrances (IFF), formerly DuPont’s Nutrition & Biosciences business.

Recently, the draft (3) and whole genome (4) sequences of *L. plantarum* ATCC 202195 were published. Knowledge of the whole genome sequence of *L. plantarum* ATCC 202195 provides an opportunity to identify sequences of DNA that are unique to *L. plantarum* ATCC 202195 and to utilize these genetic signatures for strain-level molecular detection. Since the beneficial effects of probiotics are often strain specific (5, 6), molecular methods to identify and quantify probiotic strains in human biological samples enable the ascertainment of microbiological efficacy endpoints (e.g., abundance in stool samples) and safety outcomes (e.g., probiotic-associated bacteremia). While reports are rare, probiotics can translocate and cross the gut-blood barrier as viable microorganisms and cause bacteremia and sepsis (7 – 9). In addition, the ability to detect and enumerate specific probiotic strains provides a mechanism to detect the probiotic in specimens from participants in the placebo and control arms of a study so as to be able to monitor for contamination that could potentially mask either a benefit or harm of the probiotic (10). Similarly, a tool that can discriminate between different bacterial strains and quantify the strain of interest is also important in the commercial preparation and labeling of probiotic products. For example, the FAO/WHO guidelines for the evaluation of probiotics in food products recommend that probiotic stocks should be periodically checked to confirm strain identity, and an accurate cell count should be included on product labels (11).

Real-time quantitative PCR (qPCR) is a relatively simple and low-cost technique that can be used to efficiently and reproducibly detect and quantify specific DNA sequences with a high degree of sensitivity and specificity (12 – 14). Primer and probe sequences that are specific to a species or strain of interest are required for conducting a targeted qPCR assay. Despite interest in the clinical utility of *L. plantarum* ATCC 202195, including the implementation of human clinical trials to assess safety, tolerability, and microbial outcomes (NCT05180201), primers and probes to detect and quantify this strain have yet to be published. Therefore, we designed primer and probe sequences to detect *L. plantarum* ATCC 202195 and then developed and optimized a qPCR assay to detect and quantify the strain. The ability of the primers and probe to discriminate *L. plantarum* ATCC 202195 from seven other *Lactobacillus* strains was then determined.

## RESULTS

### Primer and probe design

**Fig 1:**
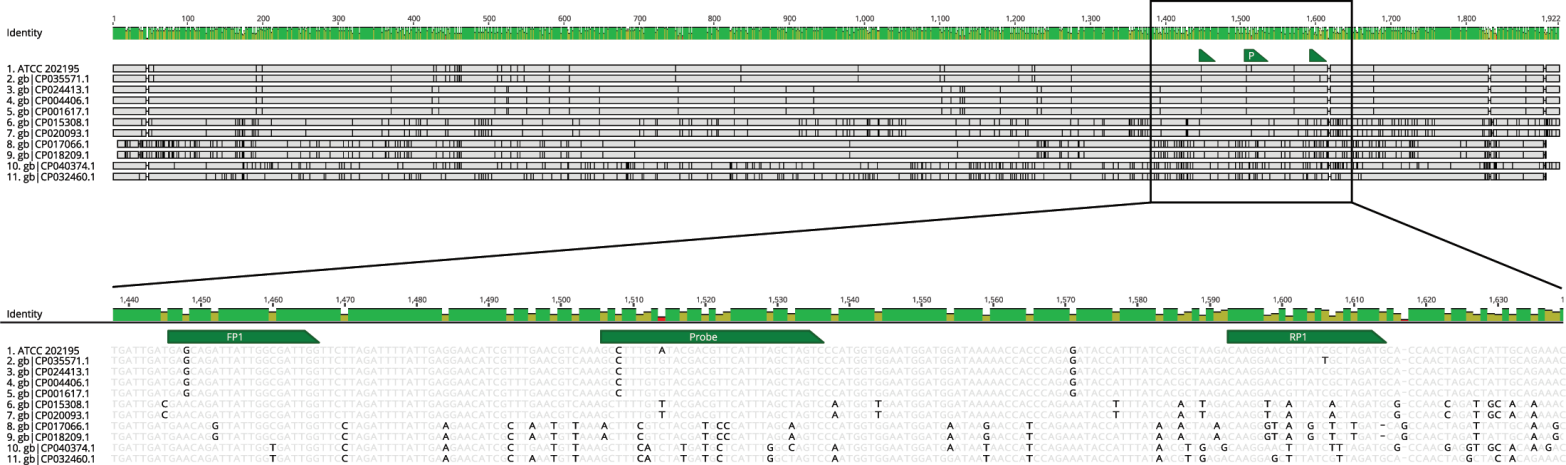
Mauve alignment of *L. plantarum* ATCC 202195 gene FEE41_08190 to the 10 most homologous sequences in NCBI. The relative height and color of the bar at the top of the figure indicate the percent identity across all 11 sequences, with green indicating high homology, yellow indicating homology in some sequences, and red indicating no homology. The black lines indicate unique base pairs. The region of the gene that is amplified using the primers and probe designed in this study is outlined with a black box and magnified. Unique base pairs are shown in bold, and the SNP in the probe sequence is highlighted in red.

While several potential single nucleotide polymorphisms (SNPs) were identified, the SNP within gene *FEE41_08190* was selected as the amplification target for *L. plantarum* ATCC 202195, as it contained few or short mononucleotide repeats, had a percentage of G+C content of less than 60%, and had the greatest variation in homology across the evaluated bacterial genomes. Gene *FEE41_08190* is annotated as a hypothetical protein that is part of the YmfN superfamily that contains phage terminase-like proteins (15, 16). While the forward and reverse primer sequences were not unique to *L. plantarum* ATCC 202195, there was no other strain that had 100% homology to both primer sequences at the time the primers were designed (Fig. 1). In contrast, the probe sequence was designed to contain a SNP that was unique to *L. plantarum* ATCC 202195 at the time of design (Fig. 1; Table 1), and the expected amplicon length was 169 bp.

**TABLE 1:** qPCR primers and probe sequences for *L. plantarum* ATCC 202195^*c*^

Target	Primer/probe name	Sequence (5′ to 3′)
*Lactiplantibacillus plantarum* ATCC 202195	LP202195-F (forward)	GAG CAG ATT ATT GGC GAT TGG
	LP202195-R (reverse)	CAT CTA GCG ATA ACG TTC CTT G
	LP202195-P (probe)	6FAM/GCC TTT GTA TAC GAC GTT CAT TTA GCT AGT C/MGB-NFQ^*a*^ ^,*b*^

^*a*^ 6FAM = 5′6-FAMTM reporter.

^*b*^ MGB-NFQ = 3′minor groove binder–non-fluorescent quencher.

^*c*^ The expected amplicon length was 169 bp, and the probe length was 31 bp.

### Linearity, efficiency, and analytical sensitivity of the *L. plantarum* ATCC 202195 qPCR assay

**Fig 2:**
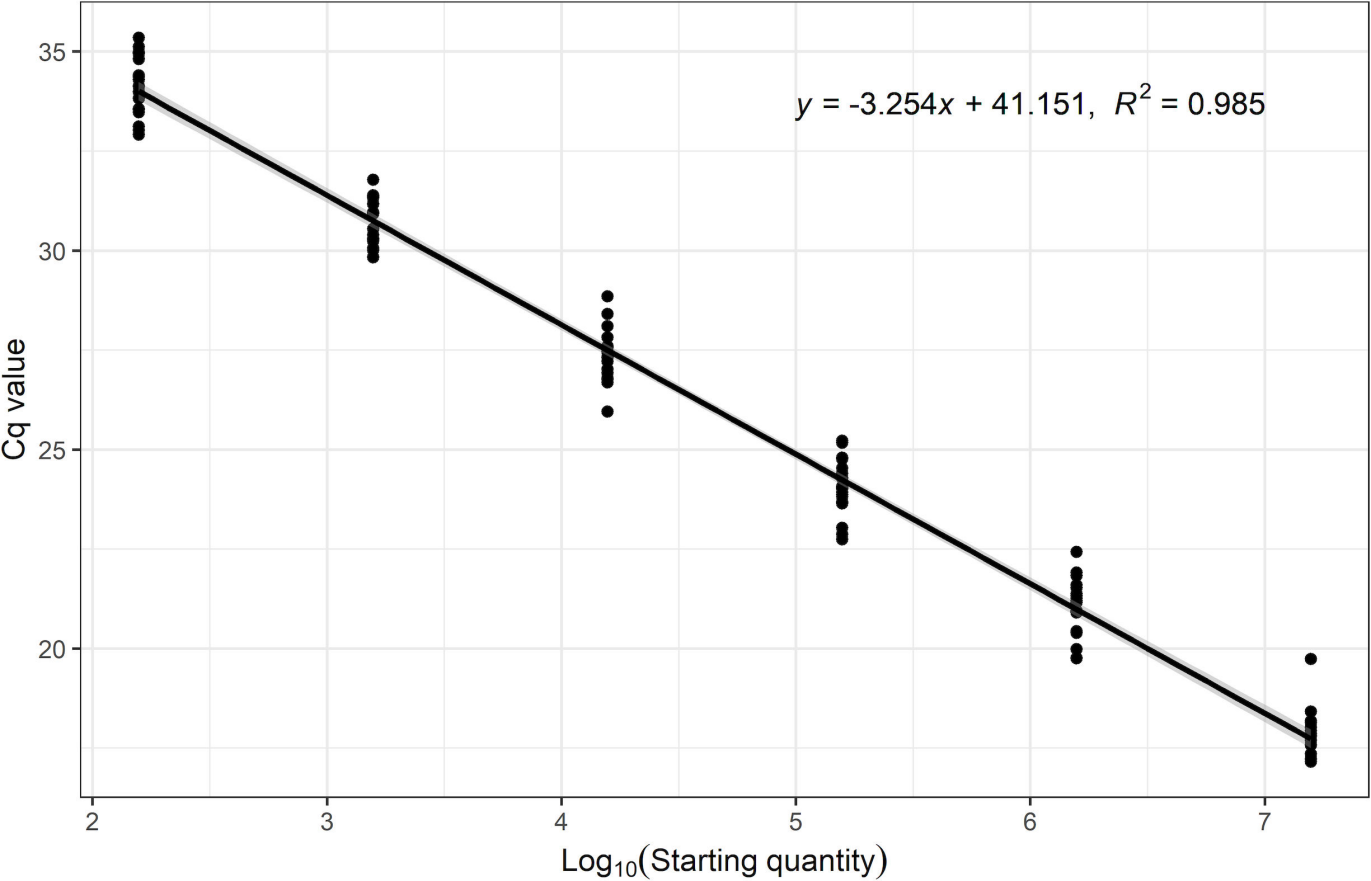
Standard curve of the qPCR assay for *L. plantarum* ATCC 202195. Cycle quantification (Cq) numbers were plotted against the log_10_ starting quantity cell numbers from six independent experiments, each including three replicates per standard. The gray shaded area represents the 95% CI of the slope of the standard curve.

**Fig 3:**
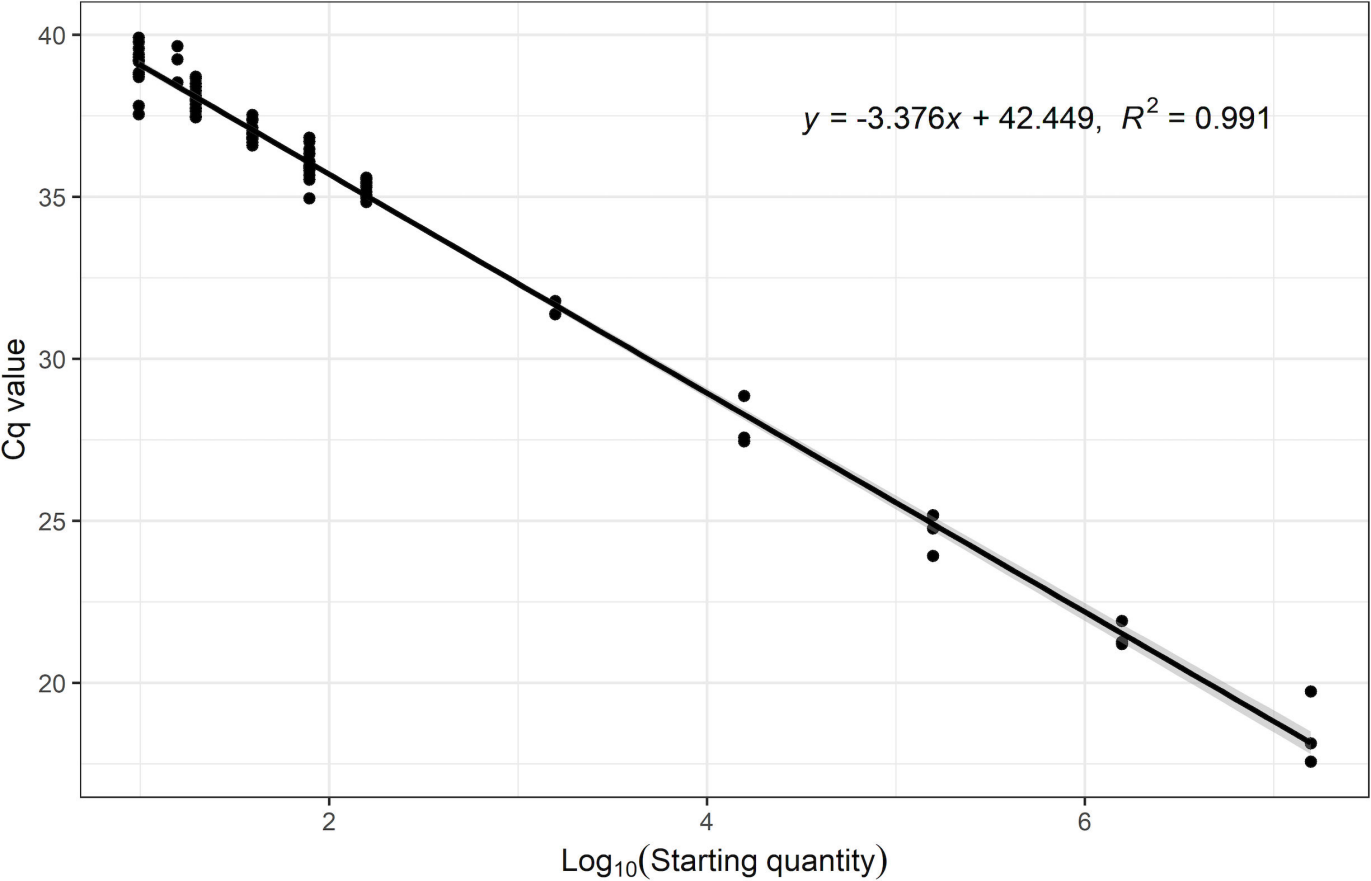
Limit of detection of the qPCR assay for *L. plantarum* ATCC 202195. Cycle quantification (Cq) numbers were plotted against the log_10_ starting quantity cell numbers from two sets of qPCR experiments, which are outlined in Table 3. The reaction efficiency was calculated to be 97.8% using the following equation, 10^(–1/slope)^ – 1, where the slope was equal to −3.3758, as determined from a linear regression model. The gray shaded area represents the 95% CI of the slope of the standard curve.

The range of the assay tested in this work was from 1.56 × 10^7^ cells per well to 1.56 × 10^2^ cells per well. Across six independent runs, the assay maintained linearity across this range of cells with a coefficient of determination (*R* ^2^) equal to or greater than 0.99 and an amplification efficiency of 97% to 110% (Table 2; Fig. 2). At 95% confidence, the limit of detection (LOD) in the assay was estimated to be approximately 15 cells (Table 3; Fig. 3).

**TABLE 2:** Coefficient of determination (*R* ^2^) values, reaction efficiencies, and standard curve equations for the qRT-PCR assay for *L. plantarum* ATCC 202195^*a*^

Run number	Standard curve equation	*R* ^2^	Amplification efficiency (%)
1	Cq = −3.183(logSQ) + 40.565	0.998	106
2	Cq = −3.096(logSQ) + 40.257	0.996	110
3	Cq = −3.177(logSQ) + 39.881	0.993	106
4	Cq = −3.34(logSQ) + 41.900	0.996	99
5	Cq = −3.329(logSQ) + 42.158	0.990	100
6	Cq = −3.398(logSQ) + 42.134	0.994	97

^*a*^ SQ, starting quantity; Cq, cycle quantification number.

**TABLE 3:** Limit of detection of the qPCR assay for *L. plantarum* ATCC 202195^*a*^

Experiment number	Cell number	Replicates run	Log (cell number)	Cq mean	Cq standard deviation	Proportion of reactions that amplified (%)	Mass of DNA input per μL
1	1.57E + 07	3	7.196	18.487	0.919	100	50 ng
	1.57E + 06	3	6.196	21.461	0.320	100	5 ng
	1.57E + 05	3	5.196	24.625	0.519	100	0.5 ng
	1.57E + 04	3	4.196	27.964	0.634	100	0.05 ng
	1,570	3	3.196	31.517	0.188	100	0.005 ng
	157	3	2.196	35.09	0.186	100	500 fg
	15.7	3	1.196	39.144	0.464	100	50 fg
	1.57	3	0.196	NaN	NaN	0	5 fg
	0.157	3	0.804	NaN	NaN	0	0.5 fg
2	157	16	2.196	35.197	0.249	100	500 fg
	78.5	16	1.895	36.036	0.442	100	250 fg
	39.25	16	1.594	36.999	0.276	100	125 fg
	19.63	16	1.293	38.029	0.377	100	62.5 fg
	9.81	16	0.992	38.999	0.696	75	31.25 fg
	4.9	16	0.69	NaN	NaN	0	15.625 fg

^*a*^ Cq, cycle quantification number; NaN, not a number.

### Intra- and inter-assay variation of the *L. plantarum* ATCC 202195 qPCR assay

The intra-assay coefficients of variation (CVs) of the cycle quantification (Cq) values ranged from 0.54% to 1.90% over six 10-fold standard dilutions (Table 4). The inter-assay CVs ranged from 0.58% to 3.72% over six 10-fold standard dilutions (Table 4). The Cq variation between assays at the lowest concentration measured was minimal [33.51 (0.43)].

**TABLE 4:** Intra- and inter-assay variation of the qPCR assay for *L. plantarum* ATCC 202195^*c*^

	Intra-assay variation				Inter-assay variation	
Cells per reaction	Cq, mean (SD)—Plate 1	Cq, mean (SD)—Plate 2	Cq, mean (SD)—Plate 3	CV^*a*^ (%)	Cq, mean (SD)	CV^*b*^ (%)
1.57 E7	17.88 (0.16)	18.05 (0.32)	17.59 (0.52)	1.895	17.84 (0.23)	1.313
1.57 E6	20.74 (0.29)	21.36 (0.22)	19.84 (0.13)	1.032	20.65 (0.77)	3.717
1.57 E5	23.96 (0.12)	23.74 (0.12)	22.89 (0.15)	0.543	23.53 (0.57)	2.405
1.57 E4	27.05 (0.15)	27.22 (0.41)	26.48 (0.45)	1.258	26.92 (0.39)	1.440
1.57 E3	30.30 (0.24)	30.24 (0.20)	29.97 (0.23)	0.749	30.17 (0.17)	0.575
1.57 E2	33.81 (0.33)	33.70 (0.25)	33.02 (0.10)	0.679	33.51 (0.43)	1.277

^*a*^ The intra-assay CV was calculated by generating the mean Cq and SD for replicates of each of the six standards within a given plate and then averaging the within-plate CVs for each standard across the three different plates that were run.

^*b*^ The inter-assay CV was calculated by determining the mean Cq value for all replicates of each of the six standards within a plate. The overall mean computed as the mean Cq per plate and SD computed as the SD of the mean Cq per plate were used to determine an inter-assay CV% for each standard.

^*c*^ CV, coefficient of variation; SD, standard deviation; Cq, cycle quantification number.

### Strain discriminating capacity of the *L. plantarum* ATCC 202195 qPCR assay

Amplification by the designed primers and probe was evident in all wells containing gDNA of *L. plantarum* ATCC 202195, including wells that contained a mixture of *L. plantarum* ATCC 202195 genomic DNA and DNA from other non-target lactobacilli (Table 5). In contrast, no amplification was observed in the reaction wells containing DNA from *L. plantarum* ATCC 14917, *Lactobacillus crispatus* VPI 3199 ATCC 33820DQ, *Lactobacillus gasseri* DSM 20243 ATCC 33323DQ, *Lactobacillus iners* AB107 ATCC 55195DQ, *Lactobacillus acidophilus* ATCC 4357D-5, *Lacticaseibacillus casei* ATCC 334D-5, and *Limosilactobacillus reuteri* F275 ATCC 23272D-5 as well as the reaction wells that contained pooled DNA of these non-target lactobacilli (Table 5). Likewise, no amplification was observed for reaction wells that contained DNA extracted from stool samples from infants without any exposure to *L. plantarum* ATCC 202195 and prior to the introduction of the probiotic into their community if those stool samples were not pre-mixed with *L. plantarum* ATCC 202195 cells (Tables 5 and 6).

**TABLE 5:** Analytical specificity of the qPCR assay for *L. plantarum* ATCC 202195

Reaction no.	Contents of reaction	Reaction contained *L. plantarum* ATCC 202195	Preparation description	Number of reactions run	Number of reactions that amplified	MeanCq^*c*^	Cq range across all reactions
1	*L. crispatus* VPI 3199 ATCC 33820DQ (2.5 ng/μL)	No	Single strain	4	0	^*d*^-	-
2	*L. crispatus* VPI 3199 ATCC 33820DQ (0.5 ng/μL)	No	Single strain	4	0	-	-
3	*L. gasseri* DSM 20243 ATCC 33323DQ (2.5 ng/μL)	No	Single strain	4	0	-	-
4	*L. gasseri* DSM 20243 ATCC 33323DQ (0.5 ng/μL)	No	Single strain	4	0	-	-
5	*L. acidophillus* ATCC 4357D-5 (2.5 ng/μL)	No	Single strain	4	0	-	-
6	*L. acidophillus* ATCC 4357D-5 (0.5 ng/μL)	No	Single strain	4	0	-	-
7	*L. casei* ATCC 334D-5 (2.5 ng/μL)	No	Single strain	4	0	-	-
8	*L. casei* ATCC 334D-5 (0.5 ng/μL)	No	Single strain	4	0	-	-
9	*L. inners* AB107 ATCC 55195DQ (2.5 ng/μL)	No	Single strain	4	0	-	-
10	*L. inners* AB107 ATCC 55195DQ (0.5 ng/μL)	No	Single strain	4	0	-	-
11	*L. reuteri* F275 ATCC 23272D-5 (2.5 ng/μL)	No	Single strain	4	0	-	-
12	*L. reuteri* F275 ATCC 23272D-5 (0.5 ng/μL)	No	Single strain	4	0	-	-
13	*L. plantarum* ATCC 14917 (50 ng/μL)	No	Single strain	3	0	-	-
14	*L. plantarum* ATCC 14917 (5 ng/μL)	No	Single strain	3	0	-	-
15	*L. plantarum* ATCC 14917 (2.5 ng/μL)	No	Single strain	2	0	-	-
16	*L. plantarum* ATCC 14917 (0.5 ng/μL)	No	Single strain	5	0	-	-
17	*L. plantarum* ATCC 202195 (50 ng/μL)	Yes	Single strain	11	11	17.98	17.16, 19.72
18	*L. plantarum* ATCC 202195 (5 ng/μL)	Yes	Single strain	11	11	21.66	20.44, 22.89
19	*L. plantarum* ATCC 202195 (0.5 ng/μL)	Yes	Single strain	11	10	24.83	24.07, 26.36
20	*L. plantarum* ATCC 202195 (0.05 ng/μL)	Yes	Single strain	11	11	28.25	27.32, 30.07
21	*L. plantarum* ATCC 202195 (0.005 ng/μL)	Yes	Single strain	11	11	31.7	30.93, 33.75
22	*L. plantarum* ATCC 202195 (0.0005 ng/μL)	Yes	Single strain	11	11	35.16	34.30, 37.05
23	*L. plantarum* ATCC 202195 plus *L. crispatus* VPI 3199 ATCC 33820DQ (1:1 mixture, 2.5 ng/μL each)	Yes	Multi-strain	4	4	23.41	22.67, 23.86
24	*L. plantarum* ATCC 202195 plus *L. crispatus* VPI 3199 ATCC 33820DQ (1:1 mixture, 0.5 ng/μL each)	Yes	Multi-strain	4	4	25.96	25.63, 26.17
25	*L. plantarum* ATCC 202195 plus *L. gasseri* DSM 20243 ATCC 33323DQ (1:1 mixture, 2.5 ng/μL each)	Yes	Multi-strain	4	4	23.18	22.59, 23.72
26	*L. plantarum* ATCC 202195 plus *L. gasseri* DSM 20243 ATCC 33323DQ (1:1 mixture, 0.5 ng/μL each)	Yes	Multi-strain	4	4	25.46	25.23, 25.75
27	*L. plantarum* ATCC 202195 plus *L. acidophillus* (1:1 mixture, 2.5 ng/μL each)	Yes	Multi-strain	4	4	23.1	22.73, 23.29
28	*L. plantarum* ATCC 202195 plus *L. acidophillus* (1:1 mixture, 0.5 ng/μL each)	Yes	Multi-strain	4	4	25.76	25.45, 26.24
29	*L. plantarum* ATCC 202195 plus *L. casei* (1:1 mixture, 2.5 ng/μL each)	Yes	Multi-strain	4	4	23.53	23.39, 23.91
30	*L. plantarum* ATCC 202195 plus *L. casei* (1:1 mixture, 0.5 ng/μL each)	Yes	Multi-strain	4	4	25.49	25.19, 25.94
31	*L. plantarum* ATCC 202195 plus *L. inners* AB107 ATCC 55195DQ (1:1 mixture, 2.5 ng/μL each)	Yes	Multi-strain	4	4	23.21	22.44, 23.80
32	*L. plantarum* ATCC 202195 plus *L. inners* AB107 ATCC 55195DQ (1:1 mixture, 0.5 ng/μL each)	Yes	Multi-strain	4	4	25.5	25.22, 26.05
33	*L. plantarum* ATCC 202195 plus *L. reuteri* (1:1 mixture, 2.5 ng/μL each)	Yes	Multi-strain	4	4	23.52	23.27, 24.10
34	*L. plantarum* ATCC 202195 plus *L. reuteri* (1:1 mixture, 0.5 ng/μL each)	Yes	Multi-strain	4	4	25.87	25.57, 26.03
35	*L. plantarum* ATCC 202195 plus *L. plantarum* ATCC 14917 (1:1 mixture, 50 ng/μL each)	Yes	Multi-strain	3	3	18.46	17.21, 19.83
36	*L. plantarum* ATCC 202195 plus *L. plantarum* ATCC 14917 (1:1 mixture, 5 ng/μL each)	Yes	Multi-strain	3	3	21.48	21.35, 21.56
37	*L. plantarum* ATCC 202195 plus *L. plantarum* ATCC 14917 (1:1 mixture, 2.5 ng/μL each)	Yes	Multi-strain	2	2	23.08	22.95, 23.22
38	*L. plantarum* ATCC 202195 plus *L. plantarum* ATCC 14917 (1:1 mixture, 0.5 ng/μL each)	Yes	Multi-strain	5	5	25.51	24.60, 26.27
39	Infant stool samples collected in an observational study conducted in Dhaka, Bangladesh	No	Stool samples	90^*a*^	0	-	-
40	Infant stool samples collected in an observational study conducted in Dhaka, Bangladesh plus *L. plantarum* ATCC 202195 cells (2.35 × 10^7^)	Yes	Stool samples	10^*b*^	10	20.62	20.02, 21.21
41	Infant stool samples collected in an observational study conducted in Dhaka, Bangladesh plus *L. plantarum* ATCC 202195 cells (2.35 × 10^6^)	Yes	Stool samples	10^*b*^	10	23.09	21.48, 24.06
42	Infant stool samples collected in an observational study conducted in Dhaka, Bangladesh plus *L. plantarum* ATCC 202195 cells (2.35 × 10^5^)	Yes	Stool samples	10^*b*^	10	27.34	26.25, 28.13

^*a*^ DNA extracted from 80 stool samples was run once, while DNA extracted from the five stool samples used in the *L. plantarum* ATCC 202195 cell spike-in assays was run in duplicate.

^*b*^ DNA extracted from the five stool samples that were mixed with *L. plantarum* ATCC 202195 cells was run in duplicate.

^*c*^ Cq, cycle quantification number.

^*d*^ ”-” signifies that data are not applicable in a given cell.

**TABLE 6:** Recovery of *L. plantarum* ATCC 202195 gDNA from stool samples spiked with *L. plantarum* ATCC 202195 cells^*b*^

Sample description	Expected log starting quantity cells	Observed log starting quantity cells, mean (SD)^*a*^	Observed log starting quantity minus expected log starting quantity	Percent recovery (observed log starting quantity divided by expected log starting quantity)	Expected Cq value	Observed Cq value, mean (SD)^*a*^	Observed Cq value minus expected Cq value
Stool sample 1 (100 mg) plus 9.42 × 10^8^ *L. plantarum* ATCC 202195 cells	7.37	7.28 (0.11)	0.10	98.8	20.39	20.71 (0.38)	−0.32
Stool sample 2 (100 mg) plus 9.42 × 10^8^ *L. plantarum* ATCC 202195 cells	7.37	7.41 (0.09)	−0.04	100.5	20.39	20.24 (0.30)	0.14
Stool sample 3 (100 mg) plus 9.42 × 10^8^ *L. plantarum* ATCC 202195 cells	7.37	7.36 (0.00)	0.01	99.9	20.39	20.43 (0.02)	−0.04
Stool sample 4 (100 mg) plus 9.42 × 10^8^ *L. plantarum* ATCC 202195 cells	7.37	7.32 (0.07)	0.05	99.3	20.39	20.55 (0.25)	−0.17
Stool sample 5 (100 mg) plus 9.42 × 10^8^ *L. plantarum* ATCC 202195 cells	7.37	7.13 (0.01)	0.24	96.7	20.39	21.19 (0.03)	−0.80
Stool sample 1 (100 mg) plus 9.42 × 10^7^ *L. plantarum* ATCC 202195 cells	6.37	7.04 (0.01)	−0.67	110.5	23.78	21.50 (0.02)	2.28
Stool sample 2 (100 mg) plus 9.42 × 10^7^ *L. plantarum* ATCC 202195 cells	6.37	6.65 (0.02)	−0.28	104.4	23.78	22.83 (0.08)	0.94
Stool sample 3 (100 mg) plus 9.42 × 10^7^ *L. plantarum* ATCC 202195 cells	6.37	6.44 (0.09)	−0.07	101.1	23.78	23.54 (0.29)	0.24
Stool sample 4 (100 mg) plus 9.42 × 10^7^ *L. plantarum* ATCC 202195 cells	6.37	6.31 (0.03)	0.07	99.1	23.78	24.00 (0.09)	−0.22
Stool sample 5 (100 mg) plus 9.42 × 10^7^ *L. plantarum* ATCC 202195 cells	6.37	6.42 (0.05)	−0.05	100.8	23.78	23.60 (0.18)	0.18
Stool sample 1 (100 mg) plus 9.42 × 10^6^ *L. plantarum* ATCC 202195 cells	5.37	5.56 (0.12)	−0.19	103.5	27.17	26.54 (0.41)	0.63
Stool sample 2 (100 mg) plus 9.42 × 10^6^ *L. plantarum* ATCC 202195 cells	5.37	5.51 (0.03)	−0.14	102.6	27.17	26.69 (0.09)	0.48
Stool sample 3 (100 mg) plus 9.42 × 10^6^ *L. plantarum* ATCC 202195 cells	5.37	5.11 (0.03)	0.26	95.2	27.17	28.05 (0.11)	−0.88
Stool sample 4 (100 mg) plus 9.42 × 10^6^ L. plantarum ATCC 202195 cells	5.37	5.14 (0.01)	0.23	95.7	27.17	27.95 (0.04)	−0.78
Stool sample 5 (100 mg) plus 9.42 × 10^6^ *L. plantarum* ATCC 202195 cells	5.37	5.28 (0.01)	0.09	98.3	27.17	27.47 (0.04)	−0.30
Stool sample 1 (100 mg) with no *L. plantarum* ATCC 202195 cells added	^*c*^-	-	-	-	-	-	-
Stool sample 2 (100 mg) with no *L. plantarum* ATCC 202195 cells added	-	-	-	-	-	-	-
Stool sample 3 (100 mg) with no *L. plantarum* ATCC 202195 cells added	-	-	-	-	-	-	-
Stool sample 4 (100 mg) with no *L. plantarum* ATCC 202195 cells added	-	-	-	-	-	-	-
Stool sample 5 (100 mg) with no *L. plantarum* ATCC 202195 cells added	-	-	-	-	-	-	-

^*a*^ Mean and SD between two replicates.

^*b*^ Cq, cycle quantification number.

^*c*^ "-” signifies that data are not applicable in a given cell.

### Recovery of *L. plantarum* ATCC 202195 gDNA from stool samples spiked with *L. plantarum* ATCC 202195 cells

When known quantities of *L. plantarum* ATCC 202195 cells were added to stool samples from infants without any exposure to *L. plantarum* ATCC 202195, amplification was observed in the extracted DNA sample (Tables 5 and 6). The difference between the observed log starting quantity and the expected log starting quantity of *L. plantarum* ATCC 202195 cells ranged from −0.67 to 0.26, and the percent recovery of *L. plantarum* ATCC 202195 gDNA in the assay ranged from 95.2% to 110.5% (Table 6).

## DISCUSSION

A probe-based qPCR assay to identify and quantify *L. plantarum* ATCC 202195 was found to have high precision, sensitivity, and specificity for the target strain. This assay is being applied to samples collected in a recent clinical trial (Clinicaltrials.gov: NCT05180201) and is suitable for use both in future research activities and in the development of commercial production pipelines. While rare, cases of sepsis associated with the use of lactobacilli-containing probiotics have been reported (17 – 20). Probiotic-associated bacteremia is commonly identified using a variety of methods, including conventional blood cultures, antibiotic susceptibility testing, and reviewing medical charts for evidence of prior probiotic exposure. However, to verify a link between the sepsis causative agent and an exogenously provided probiotic, molecular techniques must be used to compare culture isolates with the probiotic source (19). Thus, if *L. plantarum* ATCC 202195 is ever the suspected cause of bacteremia, the assay presented in this work could be adapted for use in a clinical setting to verify the molecular identity of bacterial isolates.

The assay met all criteria of an optimized qPCR assay, including a linear standard curve with an *R* ^2^ value of the regression line that is greater than 0.98, a high amplification efficiency (90% to 110%) over a wide dynamic range that is at least three orders of magnitude for gDNA (21), a demonstrated reproducibility of Cq values between replicate measurements (22), and high sensitivity and specificity for the target of interest. The assay amplified *L. plantarum* ATCC 202195 in both single-strain and multiple-strain samples without the amplification of non-specific *Lactobacillus* targets. Furthermore, there was no detectable amplification of potentially cross-reacting strains and species, including *L. plantarum* ATCC 14917, *L. crispatus* VPI 3199 ATCC 33820DQ, *L. gasseri* DSM 20243 ATCC 33323DQ, *L. iners* AB107 ATCC 55195DQ, *L. acidophilus* ATCC 4357D-5, *L. casei* ATCC 334D-5, and *L. reuteri* F275 ATCC 23272D-5.

A previous hospital-based trial that was conducted in India demonstrated that *L. plantarum* ATCC 202195 was only detected in stool samples collected from infants who were orally supplemented with a 7-day synbiotic regimen of *L. plantarum* ATCC 202195 plus FOS (2). *L. plantarum* ATCC 202195 was not detected in any stool samples collected from infant participants who were supplemented with a placebo (dextrose saline), suggesting that *L. plantarum* ATCC 202195 may not be found under natural conditions (2). Consistent with these findings, there was no amplification in any stool samples collected from infants who had not been exposed to *L. plantarum* ATCC 202195 unless the stool samples were spiked with *L. plantarum* ATCC 202195 cells. The percentage recovery of *L. plantarum* ATCC gDNA from stool samples spiked with *L. plantarum* ATCC 202195 cells was high, indicating that the assay is well suited for the ascertainment of microbiological endpoints, including the abundance of *L. plantarum* ATCC 202195 cells in stool samples.

The use of qPCR to detect and quantify *L. plantarum* ATCC 202195 offers several advantages, including the ability to determine the absolute quantity of target DNA present in an unknown sample in near real time without the need for post-PCR processing. However, the assay was not designed to differentiate viable from unviable cells, and thus, it was not possible to use this approach to estimate the abundance of active probiotics within a given sample. In addition, the amplification of *L. plantarum* ATCC 202195 from different matrices (i.e., blood) was not assessed here and warrants further investigation. Moreover, while the precision and sensitivity of the qPCR assay may have been higher if droplet digital PCR (ddPCR) had been used (23), the optimized qPCR assay had low inter- and intra-assay variation and a relatively low LOD of about 15 cells. In the future, if greater precision or a lower LOD is required, the qPCR protocol could be easily adapted for ddPCR using the same primer and probe sequences (23). In addition, while the rapid pace in which bacterial strains are isolated and sequenced provides an opportunity for discovery, it also means that strain-specific PCR assays need to be revisited frequently; the publication of new genome sequences reveals target sequences are not strain specific. In fact, in May 2020, the complete genome sequence of *L. plantarum* strain AMT74419 (GCF_012974545.1 CP052869.1) was uploaded to NCBI’s RefSeq database, approximately 12 months after the target sequence for strain-specific detection of *L. plantarum* ATCC 202195 was identified. The genome of *L. plantarum* strain AMT74419 is 99.4% identical to the genome of *L. plantarum* ATCC 202195, and it contains a gene that is 100% identical to gene *FEE41_08190* (data not shown), the amplification target that was chosen for strain-specific identification of *L. plantarum* ATCC 202195. Thus, future work may need to verify the identification of *L. plantarum* ATCC 202195 by running a multiplex PCR assay that utilizes more than one gene target. In comparing the genomes of *L. plantarum* strain AMT74419 and *L. plantarum* ATCC 202195, many SNPs are available (data not shown) that could be used to design a second set of primers for a multiplex assay. Finally, gene *FEE41_08190,* the amplification target for the identification of *L. plantarum* ATCC 202195, is located within an intact prophage. Prophages are abundant in lactobacilli (24), and complete prophages can be induced, either spontaneously or through environmental stressors, released from the bacterial genome, and infect other susceptible bacteria, either causing lysis or integrating into their genomes, thereby acting as a vehicle for horizontal gene transfer. While spontaneous induction or lysis from *L. plantarum* ATCC 202195 has not been assessed or observed in this work, bacteriophage induction is possible and could result in the transfer of gene *FEE41_08190* to other strains of bacteria or prophage-free derivatives of *L. plantarum* ATCC 202195. As noted above, future assays may need to utilize a multiplex PCR assay with more than one gene target, including at least one gene located outside of an intact prophage.

### Conclusions

This is the first report of a qPCR assay that detects and quantifies *L. plantarum* ATCC 202195, a probiotic that appears to have beneficial effects in young infants. Minimal intra- and inter-assay variation, linearity of the standard curve over a wide dynamic range, adequate reaction efficiency, the absence of non-specific amplification, and verification that *L. plantarum* ATCC 202195 can be isolated and detected at high yield from a stool matrix make this assay a suitable tool for the detection and quantification of *L. plantarum* ATCC 202195.

## MATERIALS AND METHODS

### Bacterial cells, genomic DNA, and cell growth conditions

*L. plantarum* ATCC 202195 and *L. plantarum* ATCC 14917 were purchased from the ATCC (Manassas, Virginia, USA). Genomic DNA from *Lactobacillus crispatus* strain VPI 3199 ATCC 33820DQ, *Lactobacillus gasseri* strain DSM 20243 ATCC 33323DQ, *Lactobacillus iners* strain AB107 ATCC 55195DQ, *Lactobacillus acidophilus* ATCC 4357D-5, *Lacticaseibacillus casei* ATCC 334D-5, and *Limosilactobacillus reuteri* strain F275 ATCC 23272D-5 was purchased from Cedarlane Labs (Burlington, Ontario, Canada).

*L. plantarum* ATCC 202195 and *L. plantarum* ATCC 14917 cells were cultured in De Man, Rogosa, and Sharpe (MRS) broth under anaerobic conditions at 37°C for approximately 20 hours. Prior to DNA extraction, *L. plantarum* ATCC 202195 cells were enumerated using hemocytometry. In brief, cells were subjected to two overnight growth passages in MRS broth at 37°C. A total of 100 mL of overnight culture was diluted 10 times in phosphate-buffered saline (PBS) before transferring 10 µL of cell suspension into the hemocytometer (Bright-line) and viewing the cells under ×40 magnification (Nikon Optiphot). Cells were counted from 20 different grid squares by three independent laboratory personnel before being averaged. Enumerated cell suspensions were centrifuged at 10,000 × *g* for 5 minutes, and the resulting cell pellets were stored at −80°C until DNA was extracted.

### Primer and probe design

Rapid Annotation using Subsystem Technology (RAST) (25) and SEED (26) were used to identify unique regions in the *L. plantarum* ATCC 202195 genome compared to five representative *L. plantarum* genomes. Regions that were identified as unique to *L. plantarum* ATCC 202195 were compared to all publicly available genomes in NCBI (~170 genomes in May 2019) using BLASTN (27). The genes with the highest sequence homology were downloaded, and Mauve alignments were then created using Geneious Prime (Geneious Prime 2019.1.3). Alignments were used to identify SNPs that could distinguish *L. plantarum* ATCC 202195 from other *L. plantarum* strains. Primer and probe sequences were designed using Geneious (Geneious Prime 2019.1.3) (28) based on sequence homology to a single unique SNP. To ensure that the primer and probe sequences were specific to *L. plantarum* ATCC 202195, they were initially compared *in silico*, using BLAST (27), against all genomes in NCBI (15, 16) for lactobacilli (about 2,900 genomes in 2019). Primers and probes were purchased from Life Technologies Inc. (Thermo Fisher Scientific) and are detailed in Table 1.

### DNA extraction

Genomic DNA was extracted from cultured cells of *L. plantarum* ATCC 202195 and *L. plantarum* ATCC 14917 using a modified Qiagen stool DNA extraction protocol (QIAamp Fast DNA Stool Mini Kit, QIAGEN, Germany, Catalog # 51604). In brief, pelleted cells were resuspended in 0.5 mL of InhibitEX Buffer and then mixed with 1 g of a 1:1 ratio of 0.1- and 0.5-mm ceramic beads (Omni International, 19–683, 19–684-550G). Bead beating was performed for 3 minutes at the maximum speed setting using a Mini-Beadbeater-24 (Biospec Products). The bead-cell slurry was then heated at 90°C for 10 minutes, followed by a cooling step on ice for approximately 60 seconds before adding 600 µL of Buffer AL and 25 µL of proteinase K and incubating the sample at 65°C for 15 minutes. The sample was briefly vortexed for 10 minutes into a 15-minute incubation period at 65°C. After the full 15 minutes had elapsed, the sample was subjected to centrifugation at 14,000 × *g* for 1 minute. Lysates were then mixed with 600 µL of 96%–100% ethanol, applied to the QIAamp spin column, and washed with buffers AW1 and AW2, following the manufacturer’s protocol. The columns were then incubated at room temperature for 2–3 minutes prior to adding 100 µL of elution buffer directly onto the QIAamp membrane, followed by incubating the column at room temperature for 5 minutes prior to centrifugation at 20,000 × *g* for 2 minutes. The elution step was repeated by pipetting the eluate back onto the column and centrifuging at 20,000 × *g* for 2 minutes. DNA was isolated in a final volume of 100 µL elution buffer, and the total yield was measured using a NanoDrop 2000 Spectrophotometer (Thermo Scientific). DNA was stored at −80°C prior to analysis. Extraction blanks that were treated identically to sample columns were used throughout the DNA extraction process.

DNA was extracted from human stool samples using a similar adaptation of the QIAamp Fast DNA Stool Mini Kit (QIAGEN, Germany, Catalog # 51604) protocol described above with the following exceptions: infant stool samples were thawed on ice, approximately 100–150 mg was aliquoted for extraction, and 10 µL of 10X Phocid herpes virus (PhHV) (European Virus Archive – Global (EVAg), France) was added as an internal spike-in control before adding InhibitEX buffer.

### Quantitative PCR

Real-time quantitative PCR assays were performed using a 96-well CFX 96 Touch Real Time Detection System (Bio-Rad, Hercules, CA, USA). A total reaction volume of 20 µL was used for each reaction and comprised 10 µL of iTaq Universal Probe Supermix 2X (Bio-Rad, USA, Catalog # 1725134), 1 µL each of forward and reverse primer (10 µM), 0.5 µL of TaqMan Probe (10 µM), 1.5 µL of nuclease-free water, 1 µL (5% vol/vol) DMSO, and 5 µL of DNA template. DNA amplification was performed under the following cycling parameters: 5 minutes at 95˚C, followed by 40 cycles of 15 seconds at 95˚C, and 1 minute at 55˚C. Fluorescence was measured at the end of each annealing/extension step (55°C). The annealing temperature was optimized using an initial temperature gradient PCR run from 56°C to 70°C, as well as additional runs at 55°C. Unless otherwise specified, all qPCRs were performed in triplicate on separate plates, and all plates included one to two wells without a template to serve as a negative control.

To assess the linearity and amplification efficiency of the assay, six serial dilutions of gDNA from *L. plantarum* ATCC 202195 with a 10-fold dilution factor at each step were used to generate a standard curve ranging from approximately 157 cells to 1.57 × 10^7^ cells. Each dilution was tested in triplicate in six separate plates following the qPCR protocol described above. Cq values were calculated using Bio-Rad CFX Maestro software. Standard curves were generated by plotting the log starting quantity of each dilution in cells per well against their Cq values. The slope, *R* ^2^ values, and reaction efficiencies of each standard curve were calculated using Bio-Rad CFX Maestro software. To determine the analytical sensitivity of the assay, also referred to here as the LOD, the standard of *L. plantarum* ATCC 202195 containing DNA corresponding to approximately 157 cells was used to prepare six additional twofold serial dilutions, which were subjected to 16 qPCR replicates using the abovementioned cycling parameters, with the exception that a no-template control was incorrectly not included on the plate. The initial LOD was estimated by assessing the proportion of each set of 16 replicates that demonstrated amplification. The log starting cell quantity was plotted against the Cq value, and the cell number or DNA concentration at which at least 95% of replicates were amplified was identified. To further refine the LOD, 10-fold dilutions were prepared to contain cell counts that ranged from 1.57 × 10^7^ cells to 0.157 cells per well. Each standard was run in triplicate using the abovementioned cycling parameters, and the cell number or DNA concentration at which at least 95% of replicates amplified was identified. Data from both sets of experiments were assessed in totality to determine the final LOD, and the qPCR results were combined to generate a single standard curve, even though the range of sample concentrations was run across two different qPCR experiments.

Inter-assay variation was assessed by calculating the CVs from Cq values obtained from six different 10-fold standard dilutions (1.57 × 10^7^ to 1.57 × 10^2^ cells) in three different plates. Intra-assay variation was assessed by first calculating the CVs from Cq values obtained from six standard dilutions that were each analyzed in triplicate on the same plate. A total of three different plates were run, and the CVs calculated within each given plate were then averaged to obtain the average percent CV for each standard.

To assess the specificity of the primers and probe against *L. plantarum* ATCC 202195, individual qPCRs were run using genomic DNA from *L. plantarum* ATCC 14917 (0.5 ng/µL, 2.5 ng/µL, 5 ng/µL, and 50 ng/µL), *L. crispatus* VPI 3199 ATCC 33820DQ (0.5 ng/µL and 2.5 ng/µL), *L. gasseri* DSM 20243 ATCC 33323DQ (0.5 ng/µL and 2.5 ng/µL), *L. iners* AB107 ATCC 55195DQ (0.5 ng/µL and 2.5 ng/µL), *L. acidophilus* ATCC 4357D-5 (0.5 ng/µL and 2.5 ng/µL)*, L. casei* ATCC 334D-5 (0.5 ng/µL and 2.5 ng/µL), and *L. reuteri* F275 ATCC 23272D-5 (0.5 ng/µL and 2.5 ng/µL). gDNA from each of the aforementioned strains, with the exception of *L. plantarum* ATCC 14917, was pooled in a 1:1 ratio at two different DNA concentrations (0.5 ng/µL and 2.5 ng/µL) with *L. plantarum* ATCC 202195 gDNA to evaluate assay specificity in a multi-strain preparation. Genomic DNA from *L. plantarum* ATCC 14917 was pooled in a 1:1 ratio at four different DNA concentrations (0.5 ng/µL, 2.5 ng/µL, 5 ng/µL, and 50 ng/µL) with *L. plantarum* ATCC 202195 gDNA to evaluate assay specificity in a multi-strain preparation. As an additional non-target control, total DNA extracted from 85 infant stool samples collected during an observational study (Clinicaltrials.gov: NCT04012190) in Dhaka, Bangladesh, was also assessed for amplification with the primers and probes designed to specifically detect *L. plantarum* ATCC 202195. A convenience sample of 85 stool samples was randomly selected and included samples collected between 28 May 2021 and 28 August 2021, from a total of 46 infants (22 infants contributed more than one stool sample from different collection points) who ranged in age from 0 to 60 days, and none of whom had any known exposure to *L. plantarum* ATCC 202195. Most of the stool samples analyzed (*n* = 80) were collected from infants aged 0 to 14 days. Amplification of the extract from 80 of the stool samples was assessed once per sample. Five of the stool samples were aliquoted and spiked with three different known starting quantities of *L. plantarum* ATCC 202195 cells prior to DNA extraction (9.42 × 10^8^, 9.42 × 10^7^, and 9.42 × 10^6^ cells per 100 mg of stool) such that the expected log starting quantity of cells in the qPCR well was 7.37, 6.37, and 5.37, respectively. As a negative control, one of the 100-mg stool aliquots from each of the five samples was not mixed with *L. plantarum* ATCC 202195 cells. Total DNA was extracted and assessed for amplification with the primers and probes designed against *L. plantarum* ATCC 202195 in duplicate. In all qPCR plates used to assess the specificity of the primers and probe for *L. plantarum* ATCC 202195, wells were included that contained *L. plantarum* ATCC 202195 genomic DNA across a range of cell counts (1.57 × 10^2^ to 1.57 × 10^7^ cells per well) as a positive control.
